# Global mRNA and microRNA expression dynamics in response to anthracnose infection in sorghum

**DOI:** 10.1186/s12864-020-07138-0

**Published:** 2020-11-03

**Authors:** Fuyou Fu, Gezahegn Girma, Tesfaye Mengiste

**Affiliations:** 1 Department of Botany and Plant Pathology, Purdue University, West Lafayette, IN 47907 USA; 2 Present address: Agriculture and Agri-Food Canada, Plant Gene Resources of Canada, Saskatoon Research and Development Centre, 107 Science Place, Saskatoon, SK S7N 0X2 Canada

**Keywords:** Sorghum, mRNA, miRNA, Fungal infection, anthracnose, RNA-seq, Differentially expressed genes

## Abstract

**Background:**

Anthracnose is a damaging disease of sorghum caused by the fungal pathogen *Colletotrichum sublineolum.* Genome-wide mRNA and microRNA (miRNA) profiles of resistant and susceptible sorghum genotypes were studied to understand components of immune responses, and fungal induced miRNA and target gene networks.

**Results:**

A total of 18 mRNA and 12 miRNA libraries from resistant and susceptible sorghum lines were sequenced prior to and after inoculation with *C. sublineolum*. Significant differences in transcriptomes of the susceptible and resistant genotypes were observed with dispersion distance and hierarchical cluster tree analyses. Of the total 33,032 genes predicted in the sorghum genome, 19,593 were induced by *C. sublineolum,* and 15,512 were differentially expressed (DEGs) between the two genotypes. The resistant line was marked by significant reprogramming of the transcriptome at 24 h post inoculation (hpi), and a decrease at 48 hpi, whereas the susceptible line displayed continued changes in gene expression concordant with elevated fungal growth in the susceptible genotype. DEGs encode proteins implicated in diverse functions including photosynthesis, synthesis of tetrapyrrole, carbohydrate and secondary metabolism, immune signaling, and chitin binding. Genes encoding immune receptors, MAPKs, pentatricopeptide repeat proteins, and WRKY transcription factors were induced in the resistant genotype. In a parallel miRNA profiling, the susceptible line displayed greater number of differentially expressed miRNAs than the resistant line indicative of a widespread suppression of gene expression. Interestingly, we found 75 miRNAs, including 36 novel miRNAs, which were differentially expressed in response to fungal inoculation. The expression of 50 miRNAs was significantly different between resistant and susceptible lines. Subsequently, for 35 differentially expressed miRNAs, the corresponding 149 target genes were identified. Expression of 56 target genes were significantly altered after inoculation, showing inverse expression with the corresponding miRNAs.

**Conclusions:**

We provide insights into genome wide dynamics of mRNA and miRNA profiles, biological and cellular processes underlying host responses to fungal infection in sorghum. Resistance is correlated with early transcriptional reprogramming of genes in various pathways. Fungal induced genes, miRNAs and their targets with a potential function in host responses to anthracnose were identified, opening avenues for genetic dissection of resistance mechanisms.

**Supplementary information:**

The online version contains supplementary material available at 10.1186/s12864-020-07138-0.

## Background

Sorghum (*Sorghum bicolor* (L.) Moench) is one of the most important cereal crops cultivated mainly in warm tropical regions of the world as a major source of grain and feed for livestock [1, 2]. Global annual production of sorghum has reached over 68 million tonnes from a total area of about 44 million hectares in recent years [3]. However, productivity in developing countries remains low due to diseases, parasitic weeds and abiotic factors such as poor soil fertility, erratic rainfall and low/high temperature stresses [4–6]. Sorghum anthracnose caused by the fungal pathogen, *Colletotrichum sublineolum* (Henn. ex Sacc. & Trotter) [7], is one of the most devastating diseases of the crop. Grain yield losses ranging from 36% in susceptible genotypes of sorghum [8] to as high as 86% in an inbred line under maximum disease severity were reported [9].

The pathogenesis of *C. sublineolum* and its infection related morphogenesis have been studied previously [10]. The fungus exhibits a hemibiotrophic mode of nutrition with early biotrophic phase followed by necrotrophic phase characteristics of other species in the genus. In sorghum, a typical *C. sublineolum* infection produces disease lesions that turn black producing acervuli, the asexual fruiting bodies. The conidia germinate to produce germ tubes with appressoria that attach to host tissue and form penetration pegs. The infection peg enlarges into infection vesicle, giving rise to filamentous primary hyphae that grow intracellularly and further colonize adjacent cells. The necrotrophic phase of infection is marked by secondary hyphae and its extensive growth within tissues. The infection process culminates in disease symptoms on leaves, stalk, leaf peduncle, panicle, and even seeds [11]. The molecular mechanisms of infection processes are not well understood.

The most effective method to manage anthracnose disease is using resistant varieties. Accordingly, resistance cultivars have been developed in the last decades and used for crop production [12]. In addition, genetic studies have defined loci involved in resistance to *C. sublineolum* [13–15]. However, the specific molecular and genetic components that mediate resistance to the pathogen and the plant immune response pathways are still unknown. Generally, resistance to plant diseases caused by hemi-biotrophic fungi is controlled by both quantitative and simply inherited resistance mechanisms [16]. Multiple loci are defined for resistance to sorghum anthracnose [17–20]. However, whether these loci mediate effector triggered immunity or confer quantitative resistance is unclear. In all cases, neither the components of the host nor the pathogen derived elicitors are known. Similarly, our understanding of systemic resistances mediated by the plant hormones which have been well described in model systems is completely lacking. Recently, however, genome wide transcriptome and metabolic profiling studies have helped decipher host genes, pathways and cellular processes that are repressed or increased in response to infection [21, 22].

*C. sublineolum* induces metabolic changes, composed of defense-related compounds [22]. These defense active metabolites are produced from the phenylpropanoid and flavonoid pathways. Phenolic compounds, particularly antifungal 3-deoxyanthocynidin phytoalexins, apigeninidin, luteolinidin, and related conjugates were produced which supported decades old data on the contributions of these compounds to *C. sublineolum* resistance. These data are also complemented by gene expression and genetic data. The sorghum *YELLOW SEED1* which regulates the accumulation of some of these compounds have been implicated in resistance to *C. sublineolum* and other fungal pathogens [23, 24]. Gene expression data and analyses of secondary metabolites implicate genes in the PAL and flavonoid pathways for *C. sublineolum* resistance [25]. These studies defined the functional readouts of upstream signaling pathways which ultimately define factors important to restrict fungal growth and symptom development.

Small RNAs are short (approximately 18–30 nucleotides), non-coding RNA molecules that are known to regulate gene expression through post-transcriptional gene silencing, either through RNA degradation or inhibition of translation [26]. Small RNAs have been implicated in regulation of various biological process [27] including regulation of plant development, growth and response to biotic and abiotic stresses [28, 29], and other physiological roles [30]. Small RNAs are also implicated in maintenance of genome stability and induction of phenotype variation in allopolyploidisation between *Brassica* species [31]. In addition, trafficking of pathogen derived small RNAs into host cells and the subsequent suppression of immune responses have been documented [32, 33].

Changes in gene expression is one of the most rapid host responses early after infection and could suggest critical genetic players in resistance or susceptibility to infection. A battery of defense responses are transcriptionally regulated resulting in the accumulation of PR proteins, secondary metabolites and signaling components. Genome wide transcriptome profiling of sorghum has been described in previous studies [34–36]. However, genome wide transcriptome analyses, dynamics of miRNA, the regulatory role of miRNA and their expression profiles in response to *C. sublineolum* have not been studied. These is in contrast to a great number of miRNAs identified in Arabidopsis [37], rice [38], maize [39] and many other plants under various conditions. Recently, sorghum miRNAs were identified from profiling experiments in drought stressed tissues [40]. Identification and validation of miRNAs and their corresponding targets may help identify regulatory mechanisms in host resistance to sorghum anthracnose.

In the current study, we analyzed genome wide mRNA and miRNA expression profiles of resistant and susceptible sorghum genotypes in response to *C. sublineolum*. The distinct diseases responses of the two genotypes was confirmed by the extent of fungal sequences recovered in the susceptible but not in the resistant cultivar. Expression of diverse genes implicated in the biosynthesis of secondary metabolites, defense signaling pathways, and photosynthesis were altered in response to the fungus. In particular, contrasting gene expression profiles were documented in diverse functional categories such as photosynthesis, tetrapyrrole, carbohydrate binding, and secondary metabolism. Plant defense genes, components of plant pathogen recognition complexes, transcription factors, and MAPKs were upregulated in response to inoculation with distinct profiles in resistant and susceptible lines. Interestingly, the resistant genotype SC283 displayed significant changes in gene expression early after inoculation coincident with the appearance of HR but with a significant drop at 2 dpi. Furthermore, genes, miRNAs and their corresponding target genes with a potential regulatory role in sorghum immune responses were identified. Our observations pave the way for future studies on the regulatory role of miRNA, their target genes, and DEGs with function in host responses to fungal infection. This study uncovered biological and cellular processes activated in response to *C. sublineolum* that likely underpin differences in resistance or susceptibility to sorghum anthracnose.

## Results

### Responses of sorghum lines SC283 and TAM428 to *Colletotrichum sublineolum* inoculation

**Fig. 1:**
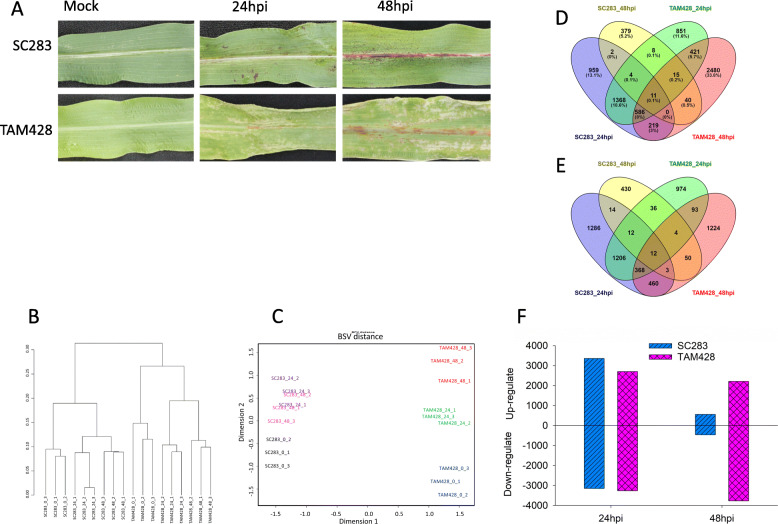
Disease response phenotypes and global evaluation of the RNA-seq data in sorghum lines SC283 and TAM428. **a** Phenotypes of SC283 and TAM428 after inoculation with *Colletotrichum sublineolum.* **b** Multidimensional scaling plot of RNA-seq data from sorghum samples. The distance on the plot shows biological coefficient of variation (BCV) between each pair of samples, and (**c**) Hierarchical clustering analysis showing relationship among samples based on RNA-seq data. **d** and (**e**) Venn diagram showing down- and up-regulated DEGs in SC283 and TAM428, respectively, (**f**) Counts of down- and up-regulated genes in SC283 and TAM428, respectively

RNA-seq was conducted to better understand the genome wide gene expression profile underlying broad-spectrum plant resistance and susceptibility in two sorghum lines, SC283 and TAM428, identified for resistance and susceptibility to multiple strains of *C. sublineolum*, respectively [41]. At 48 h after *Cs* inoculation, successful infection or resistance is visible by the early appearance of disease symptoms or the hypersensitive cell death phenotypes. In order to profile basal gene expression and early events at or before the first visible symptoms, RNA-seq was conducted on RNA samples from TAM428 and SC283 plants immediately after mock inoculation, as well as 24 and 48 h after *C. sublineolum* inoculation. Early plant responses to *C. sublineolum* inoculation appeared at 24 and 48 h post-inoculation (hpi). Hypersensitive cell death responses were observed in SC283 at 24 hpi while TAM428 displayed chlorotic patches on leaves with some reddish-brown spots on veins (Fig. 1a). In TAM428, large sectors of necrotic areas in leaf tissues were observed revealing pathogen induced cell death at 48 hpi (Fig. 1a).

### RNA-seq data reveal differential gene expression in SC283 and TAM428

RNA was isolated from leaf samples of SC283 and TAM428 sorghum genotypes that were mock, or at 24 and 48 h post inoculation (hpi) (Fig. 1a). A total of 734,963,453 high quality RNA-seq reads (average length = 99 bp) were generated by sequencing 18 RNA-seq libraries corresponding to the different samples. Each sample and each biological condition was represented by an average of 40 million and 120 million reads, respectively (Table 1). After removing adapters and low-quality reads with average Phred score below 30, the clean data of each sample were mapped against sorghum and *C. sublineolum* whole genome sequences. For each sample, more than 90% of the reads could be uniquely mapped to the sorghum reference genome. RNA reads from TAM428 samples also mapped to the *C. sublineolum* reference genome. The number of fungal reads that mapped to the *C. sublineolum* reference genome increased with an average of 0.3 and 8.1% mapped reads at 24 and 48 hpi, respectively in TAM428 (Table 1). This data shows that TAM428 supported increased fungal growth but SC283 restricted fungal proliferation. Further, hierarchical clustering analysis of the sequence data across samples indicated that SC283 and TAM428 form distinct groups (Fig. 1b). Likewise, dispersion analysis showed that the transcriptomes of SC283 and TAM428 were clearly different from each other. Based on dispersion distance, the gene expression profile of SC283 did not significantly change between 24 and 48 hpi in contrast to the expression profile in TAM428 which significantly changed between these time points (Fig. 1c) likely due to massive changes in disease symptom and fungal growth in TAM428 but not in SC283. The results were consistent with the extent of disease symptom and fungal growth in SC283 and TAM428.

**Table 1:** Sequencing metrics of the 18 RNA-seq libraries

Genotypes	Treatment	Library	Total paired reads	Mapping to sorghum	Mapping to *C. sublineolum*
SC283	0H	SC283–0-1	44,696,003	43,447,416(94.2%)	8006 (0.0%)
		SC283–0-2	44,603,755	43,369,374 (94.1%)	12,066 (0.0%)
		SC283–0-3	49,240,285	48,017,854 (94.6%)	63,971 (0.1%)
		total	138,540,043	134,834,644 (94.3%)	84,043 (0.0%)
	24H	SC283–24-1	42,612,593	40,937,987 (92.6%)	76,168 (0.2%)
		SC283–24-2	40,790,385	39,287,579 (93.3%)	56,123 (0.1%)
		SC283–24-3	36,778,762	35,867,807 (94.2%)	47,531 (0.1%)
		total	120,181,740	116,093,373 (93.4%)	179,822 (0.1%)
	48H	SC283–48-1	38,087,215	36,554,560 (93.1%)	8057 (0.0%)
		SC283–48-2	39,052,074	37,430,357 (93.5%)	167,268 (0.4%)
		SC283–48-3	38,609,348	37,148,472 (93.0%)	5634 (0.0%)
		total	115,748,637	111,133,389 (93.2%)	180,959 (0.1%)
TAM428	0H	TAM428–0-1	43,196,291	42,320,251 (95.4%)	7799 (0.0%)
		TAM428–0-2	41,822,181	41,008,508 (95.3%)	2257 (0.0%)
		TAM428–0-3	45,624,005	44,625,471 (94.2%)	1677 (0.0%)
		total	130,642,477	127,954,230 (95.0%)	11,733 (0.0%)
	24H	TAM428–24-1	37,771,230	36,513,716 (93.4%)	279,542 (0.7%)
		TAM428–24-2	38,967,041	38,086,627 (95.3%)	29,680 (0.1%)
		TAM428–24-3	38,050,166	37,120,324 (95.1%)	46,151 (0.1%)
		total	114,788,437	111,720,667 (94.6%)	355,373 (0.3%)
	48H	TAM428–48-1	35,565,060	33,495,192 (89.9%)	964,049 (2.7%)
		TAM428–48-2	40,030,840	35,770,399 (87.4%)	2,812,493 (7.0%)
		TAM428–48-3	39,466,219	31,793,828 (78.3%)	5,775,934 (14.6%)
		total	115,062,119	101,059,419 (85.2%)	9,552,476 (8.1%)

### Sorghum genes differentially expressed in response to *Colletotrichum sublineolum*

Transcriptional changes in responses to infection is an active and rapid component of plant immune response. In the present study, genes with differential expression between susceptible and resistance plants (FDR < 0.05 and *P* value < 0.01) were defined. In total, 19,593 of the 33,032 sorghum genes showed altered expression in response to *C. sublineolum*. Among them, 15,512 differentially expressed genes (DEGs) were detected in both genotypes with ≥2-fold change (FDR < 0.05 and P value < 0.01) at least in one of the treatments (Fig. 1 d, e), including 6171 up-regulated and 9341 down-regulated DEGs. The number of up-regulated genes in TAM428 (4441) were more than in SC283 (3880). Similarly, TAM428 displayed 6002 down-regulated genes which was more than the number of genes down-regulated in SC283 (3589). In particular, the number of DEGs at 48 hpi in TAM428 were significantly more than those in SC283, almost five times the number of up- and down-regulated DEGs in SC283 (Fig. 1f). These results likely reflect fungal growth in TAM428 and massive induction and activation of stress responsive genes whereas SC283 could hardly be infected. There were greater changes in gene expression at 24 hpi in SC283 with a rapid drop at 48 hpi. In order to confirm our RNA-seq data, 12 DEGs were selected for Quantitative RT-PCR. The expression patterns observed in the RNA-seq and qRT-PCR assay were largely the same (Figure S1) which demonstrated the reliability of the data produced through RNA-seq in this study.

### Functional classifications of genes differentially expressed in response to *Colletotrichum sublineolum*

**Fig. 2:**
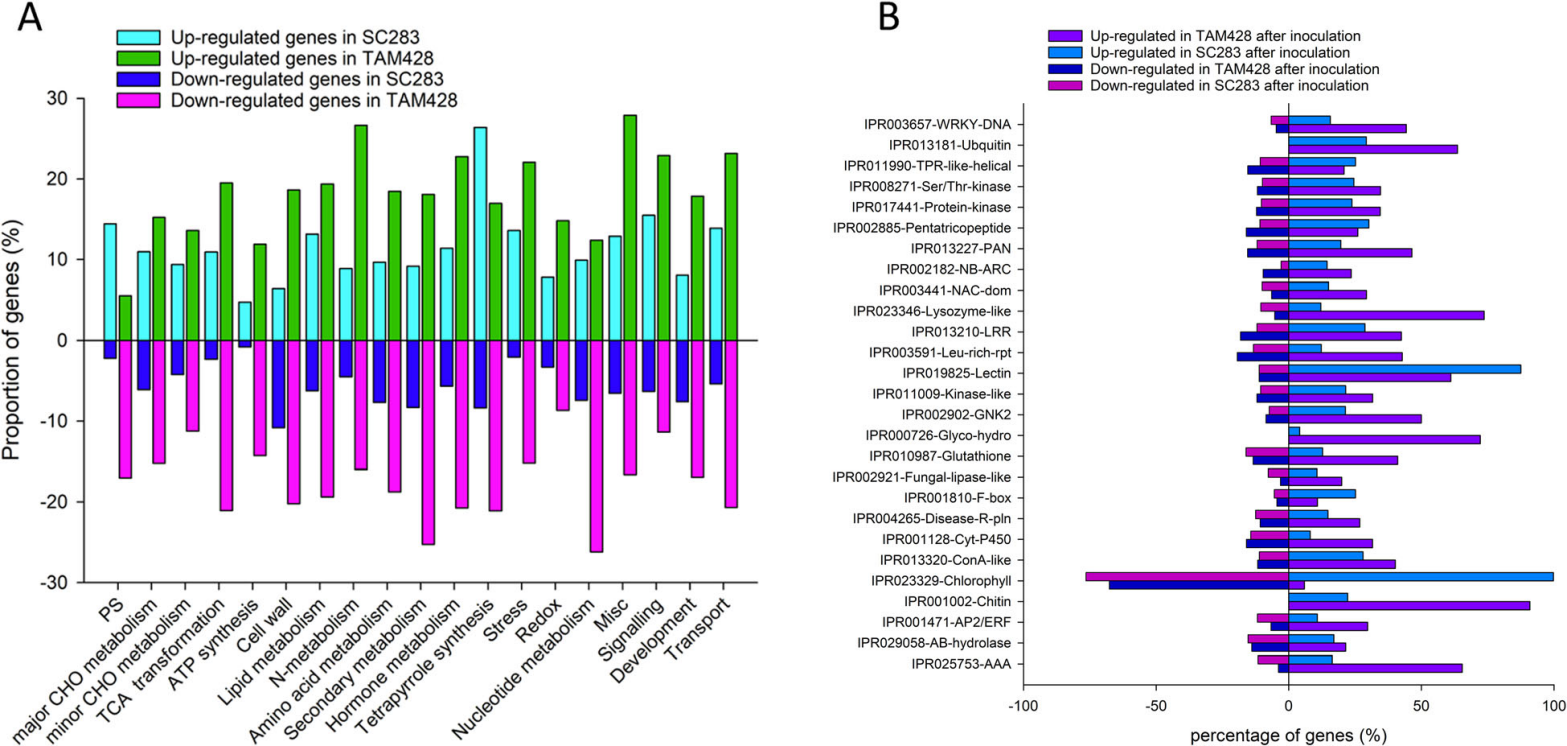
Overview of genes differentially expressed in response to *Colletotrichum sublineolum* in sorghum. **a** Functional categories for DEGs after *C. sublineolum* inoculation. Percentage of upregulated and downregulated genes are shown for selected overrepresented functional categories. Only functional categories with significant changes are displayed. CHO, carbohydrates; OPP, oxidative pentose phosphate pathway; TCA, tricarboxylic acid cycle; PS, photosynthesis. **b** Domain enrichment analysis of proteins encoded by genes differentially expressed in response to *C. sublineolum*

In order to understand the possible biological function of genes differentially expressed in the two sorghum lines, the functional classifications of DEGs (15,512) were analyzed using the InterPro database. Enrichment analysis revealed specific biological processes, protein families and metabolic pathways which were differentially represented in *C. sublineolum* inoculated sorghum genotypes (Fig. 2a). For each functional category, there were greater proportion of down-regulated functional groups in TAM428 than in SC283. However, the proportion of *C. sublineolum* up-regulated photosynthesis and tetrapyrrole synthesis genes in SC283 were higher than those in TAM428 (Fig. 2a). The proportion of down-regulated protein domains/families were not different between TAM428 and SC283 (Fig. 2b). By contrast, the proportion of up-regulated protein domain/families was significantly different between TAM428 and SC283. The proportion of up-regulated genes encoding protein domains IPR019825 (Lectin) and IPR023329 (Chlorophyll) in SC283 were more than those in TAM428. Conversely, the up-regulated genes encoding WRKY (IPR003657), Lysozyme-like (IPR023346), Glyco-hydro (IPR000726), Chitin (IPR 01002), and IPR 025753 (AAA-ATPase) in TAM428 were more than those in SC283 (Fig. 2b). These results revealed diverse processes and protein families with different functions were involved in regulating sorghum responses to *C. sublineolum*. Likewise, functional categories and overview of metabolic changes in infected leaves after inoculation was visualized with Mapman (Figure S2). Overall, the RNA-seq data suggest the early upregulation of selected genes important from photosynthesis and pathogen recognitions contributes to resistance.

### Expression of plant pattern recognition and intracellular receptor genes in response to *Colletotrichum sublineolum*

Plant receptor proteins are pathogen recognition and signaling components that determine the activation of immune responses. These molecules are primary determinants of immune response activation. The kinetics and intensity of immune response activation determine whether the pathogen colonizes the plant or is restricted at early stages of infection. Broadly, plant receptors belong to surface localized receptor-like kinase (RLKs) families with extracellular leucine-rich repeat (LRRs) domains or are nucleotide binding site and leucine rich repeat (NBS-LRR) proteins that serve as intracellular receptors. We identified a total of 1264 RLKs proteins with Pkinase (PF00069) or Pkinase_Tyr (PF07714) domains at their C-termini and LRR receptor domains in their N-termini. The RLKs were categorized into subfamilies based on their N-terminal domains. In the current study, 252 RLKs with LRR domain (PF08263 and PF00560), 77 Lectin_legB (PF00139), 76 S-locus kinase with S_locus_glycop (PF00954), 4 LysM kinase with LysM (PF01476), 132 WAKs with WAK-related domains (PF07645 or PF07714), 2 PR5K with Thaumatin (PF00314), 337 NBS-LRR proteins with NB-ARC (PF00931), and 44 CRKs with stress-antifungal (PF01657) domains were identified from sorghum genome. NBS-LRR proteins are the main class of disease resistance genes in plants and the numbers so far discovered varies across plant species. Among the 1264 RLKs proteins, 18 RLKs in SC283 and 24 RLKs in TAM428 were induced with a LogFC above 5 (32 folds change), at least once in each treatment (Table S1). Of all the RLKs, 10 were uniquely induced in SC283 and 11 induced in TAM428 and seven genes were induced in both SC283 and TAM428.

A total of 125 NBs-LRR genes were differentially expressed in response to *C. sublineolum* inoculation in TAM428 and SC283 (> 2-fod change). The number of DEGs at 24 hpi were comparable in the two genotypes, with 79 DEGs in SC283 and 73 in TAM428. respectively. However, the DEGs were significantly different at 48 hpi between SC283 and TAM428. Interestingly, only one NBs-LRR gene was down-regulated 4-fold at 48 hpi in SC283. In total, 35 NBS-LRR genes were identified at 48 hpi in TAM428, including 19 down-regulated and 16 up-regulated DEGs (Table S2). Thus, a greater number of resistance genes showed reduced expression in responses to *C. sublineolum* in the susceptible genotype.

### Defense-related genes regulated during *Colletotrichum sublineolum* infection

A battery of pathogenesis related proteins (PRs) have been studied and used as markers for activation of defense gene expression. The direct contribution of PR proteins in plant defense is still unclear due to lack of genetic evidence but are often used as markers for the activation of immune responses. Among these, chitinases are involved in the degradation of chitin, which is the primary structural component of fungal cell walls. A total of 24 sorghum putative chitinases were up-regulated in response to *C. sublineolum*. The proportion of up-regulated chitinase in TAM428 was more than those in SC283 (Fig. 2b and Table S3). Interestingly, we did not identify any differentially expressed chitinases at 48 hpi in SC283. These results may reflect the limited fungal growth in SC283 at 48 hpi.

### Sorghum WRKYs transcription factors differentially expressed in response to *Colletotrichum sublineolum*

WRKY proteins are plant specific transcription factors known for their function in plant defense by acting downstream of many immune response pathways [42]. We identified a total of 95 WRKY genes in the sorghum reference genome version 3.1. Of these, 54 WRKY genes were induced in SC283 and TAM428, including WRKY11, 28, 33, 40, 69, and 75 (Table S4). In SC283, a total of 24 WRKY genes were up-regulated at 24 hpi and 2 WRKY at 48 hpi. Similarly, in TAM428, a total of 31 and 30 WRKY genes were up-regulated at 24 and 48 hpi, respectively. Four WRKY33 homologs were identified, one of them, Sobic.009G171600, was up-regulated 8-fold at 24 hpi in SC283 and 16-fold in TAM428. WRKY33 has been implicated in resistance to both fungal and bacterial pathogens [43]. In addition, four WRKY70 genes were identified. WRKY70 is an activator of SA-induced genes and a repressor of JA-responsive genes [44]. One of the sorghum WRKY70 genes (Sobic.001G381300), was up-regulated 8-fold at 24 hpi in TAM428. Expression of *WRKY70* was increased significantly after *C. sublineolum* inoculation in SC283. These results suggest that the SA signaling pathway is activated in response to *C. sublineolum* infection and higher increase in TAM428 may indicate the differential fungal accumulation and hence activation of these genes in TAM428 background. In addition, our data suggest WRKY genes may play both negative and positive regulatory roles in different plant systems.

### Expression of MAPK pathway genes during *Colletotrichum sublineolum* infection

The MAPK pathway is central to plant immune signaling based on studies in model systems [45]. In sorghum, a total of 119 MAPK pathway genes were identified in the reference genome version 3.1. In total, 44 MAPK genes were induced during *C. sublineolum* in SC283 and TAM428, including 15 MAPKs, 6 MAPKKK, 3 MKK, 2 MK, and 5 MPK (Table S5). MAPK cascade genes were induced at 24 hpi but not differentially expressed at 48 hpi in SC283. Sobic.009G217500, MAPKKK15, was up-regulated about 10-fold at 24 hpi in SC283. Differentially expressed MAPK genes were identified both at 24 hpi and 48 hpi in TAM428. The most differentially expressed gene, Sobic.003G268800 encoding a putative MAPKKK17 was up-regulated about 16-fold (Table S5).

### Expression of Pentatricopeptide repeat genes in response to *Colletotrichum sublineolum*

PPRs are protein families implicated in various biological processes including defense [46, 47]. We identified 265 PPRs in the sorghum reference genome version 3.1. Among these, 145 PPRs were induced more than 2-fold in response to *C. sublineolum* inoculation in SC283 and TAM428 (Table S6). The most differentially expressed PPRs were identified from SC283 at 24 hpi (119 PPRs at 24 hpi and 6 PPRs at 48 hpi) and 80 PPRs at 24 hpi and 37 PPRs at 48 hpi in TAM428, respectively. Particularly, only six PPRs were down-regulated at 48 hpi in SC283. The expression level of all 37 PPRs was down-regulated at 48 hpi in TAM428. The early activation of PRRs specially, in SC283 suggest that PPRs might be involved in defense against *C. sublineolum* infection in sorghum although the mechanisms are unclear.

### Identification of sorghum miRNAs regulated by *Colletotrichum sublineolum* infection

**Fig. 3:**
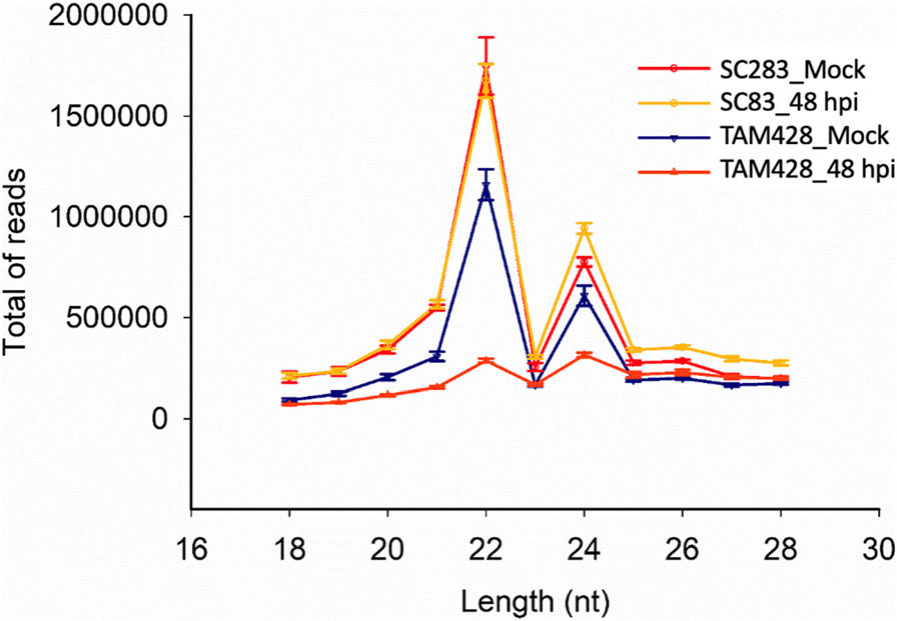
Length distributions of unique small RNAs in sorghum lines SC283 and TAM428

miRNAs are 20–24 nucleotide long non-coding RNAs abundant in plants and animals [48] with regulatory role in plant-environment interactions [26]. To identify miRNAs that may modulate responses to anthracnose, twelve small RNA libraries constructed from mock and fungal inoculated plants were sequenced, generating about 200 million small RNA reads. After low quality, rRNA and tRNAs were removed, 46 million 18 to 28 nt long small RNA reads were obtained. The size distribution of unique small RNA reads, analysis pipeline with prediction results were summarized in Fig. 3 and Figure S3. The small RNA reads were higher for both pathogen-inoculated and mock-inoculated SC283 samples. TAM428 generated lower number of small RNA reads prior to infection with further decrease at 48 hpi. This is in clear contrast to the mRNA expression profiling which generally produced a greater number of genes with altered gene expression in TAM428. Regardless of the genotype and infection, the size distribution of small RNAs peaked at 22 and 24 nt. In SC283, 91 and 153 miRNAs were identified after mock or anthracnose inoculation, respectively, suggesting increased expression of miRNAs in response to the pathogen. By contrast, in TAM428, 111, and 59 miRNAs were identified in mock and infected tissues, respectively, at 48 hpi suggesting suppression of miRNAs (Figure S3). These miRNAs included 69 known mature sorghum miRNA sequences of 241 previously annotated miRNA using miRBase version 21. A total of 80 novel mature miRNAs were generated from 123 precursors which were compared against miRBase version 21 to identify sequence homology (Table S7).

### Differential expression of sorghum miRNAs in response to *Colletotrichum sublineolum*

**Fig. 4:**
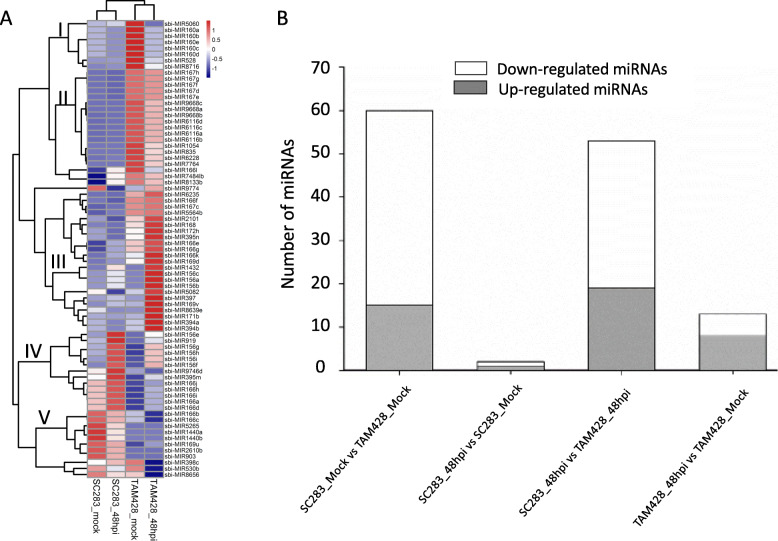
Expression profiling of miRNAs in response to *Colletotrichum sublineolum*. **a** Hierarchical clustering of differentially expressed miRNAs. The RNA samples are from mock or pathogen inoculated leaf tissues of SC283 and TAM428. Red and blue colors show higher and lower differential expression levels, respectively. The original expression values of the miRNAs were normalized using Z-score. The signal intensity ranges from − 1 to 1, as the corresponding color also changes from blue to red, and (**b**) Number of differentially expressed miRNAs. S0, mock inoculated SC283; S4, SC283 at 48 hpi; T0, mock inoculated TAM428; and T4, TAM428 at 48 hpi

To identify miRNAs associated with resistance or susceptible responses to *C. sublineolum*, differential miRNA expression was analyzed by EdgeR package [49] in R [50]. In total, 75 miRNAs showed differential expression pattern in twelve libraries with fold changes ≥2 (FDR < 0.05 and *P* value < 0.01) in at least one treatment, including 39 known miRNAs and 36 novel miRNAs (Table S7). As presented in Fig. 4a, these differentially expressed miRNAs produced five clusters that show (1) higher expression in mock inoculated TAM428, 2) higher expression after mock inoculation and at 48 hpi in TAM428, 3) higher expression in TAM428 at 48 hpi, (4) higher expression in SC283 at 48 hpi, and 5) higher expression after mock inoculation and at 48 hpi in SC283 (Fig. 4a). Interestingly, the expression of more than 50 miRNAs were significantly different between mock and fungal inoculated SC283 and TAM428 (Fig. 4b, Table S7). These miRNAs may have a role in regulating host responses to anthracnose.

### Sorghum miRNA target genes responding to *Colletotrichum sublineolum*

**Fig. 5:**
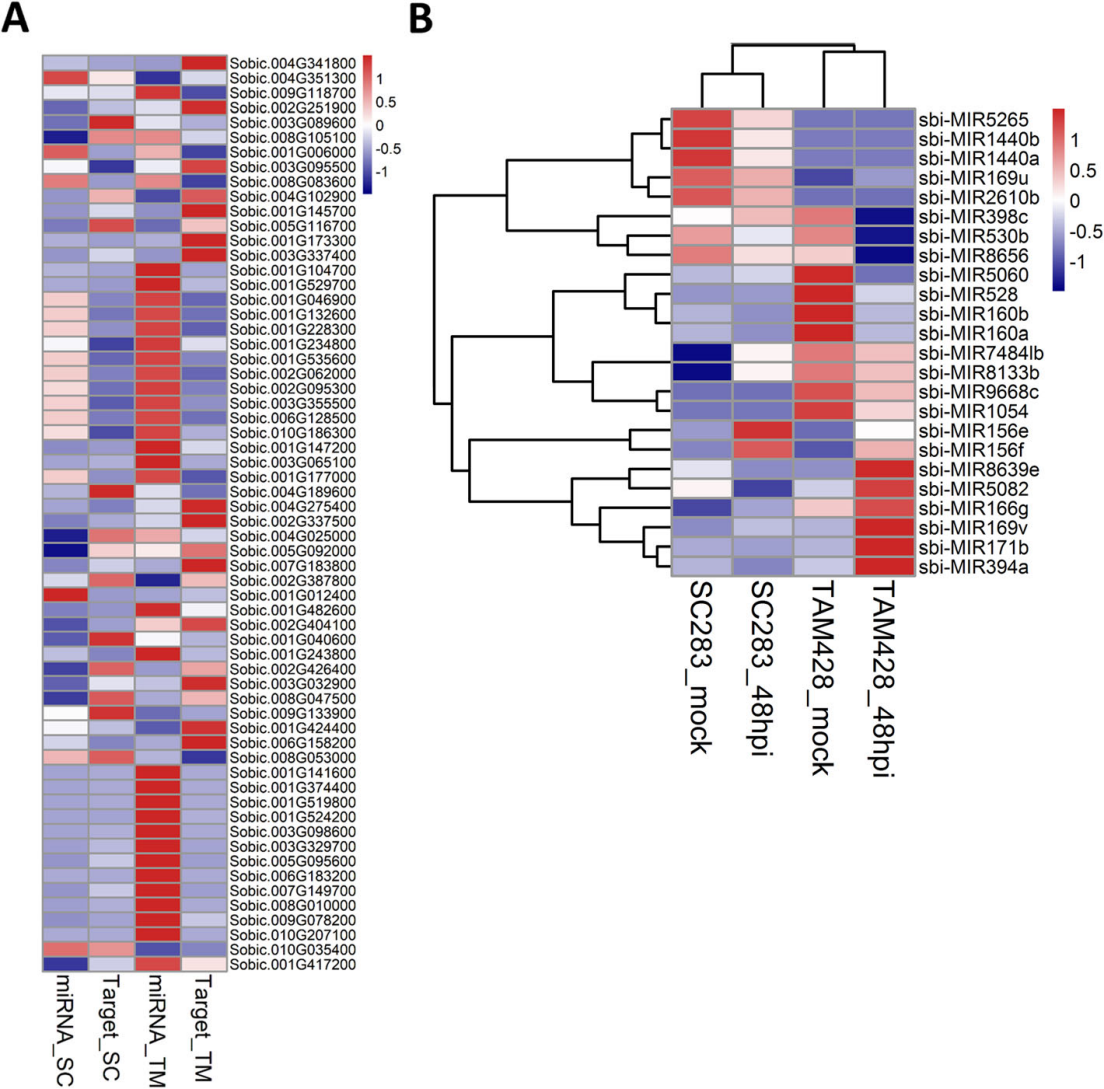
Expression of fungal regulated miRNAs and their target genes. Expression level of miRNAs (**a**) and target genes (**b**). The original expression values of miRNAs and their target genes were normalized by Z-score

**Fig. 6:**
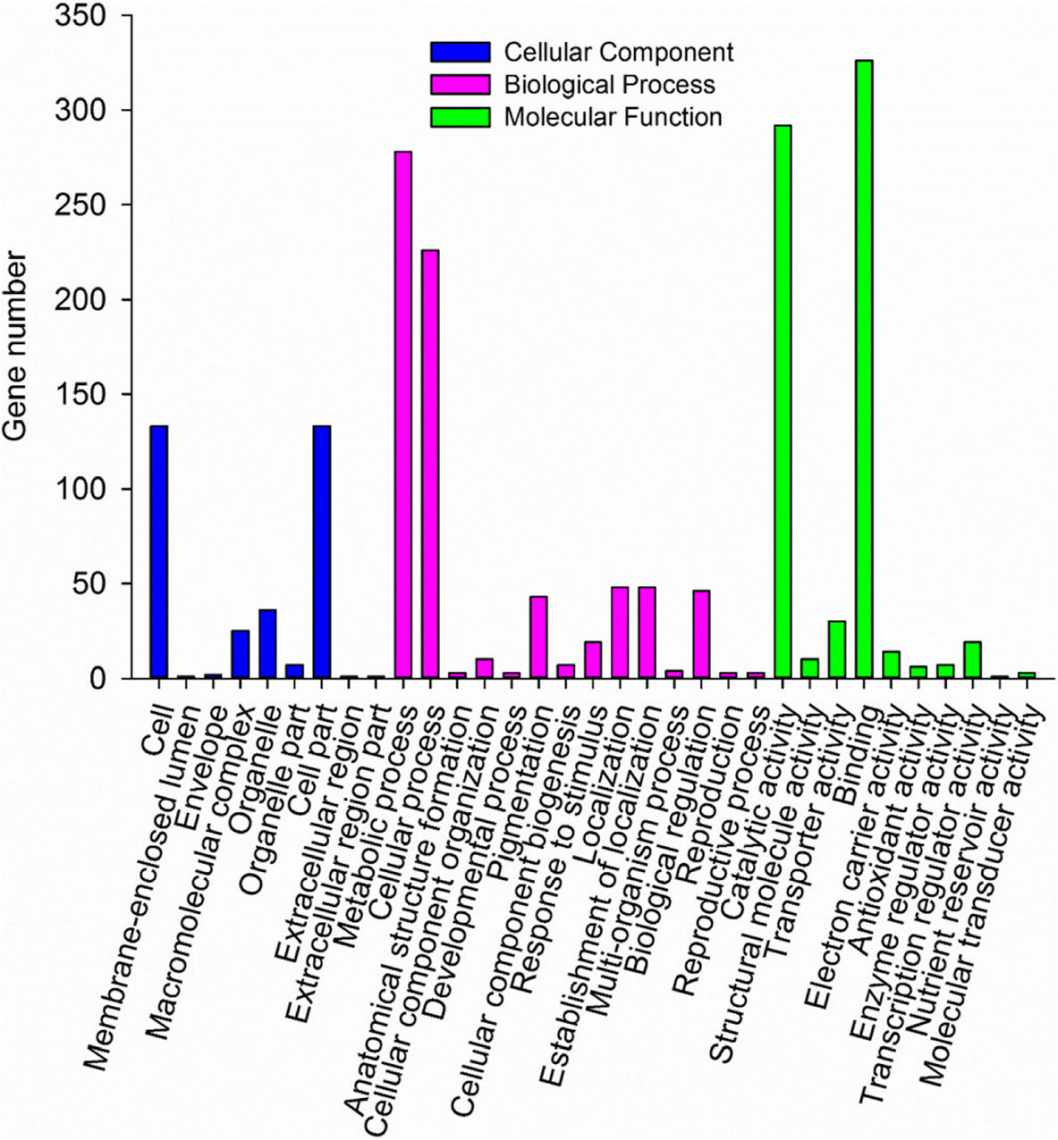
Gene ontology based functional classification of identified microRNA target genes. Blue bar represents functional category of cellular components, pink bar shows functional category of biological process and green bar indicates functional category of molecular function

miRNAs regulate gene expression mainly through post-transcriptional gene silencing [51]. The miRNAs bind to target mRNAs sequences and promote the degradation and/or inhibit the translation of their target mRNA, typically resulting in repressed gene expression. In sorghum, target gene identification by miRNAs are limited to a study of target predictions in drought-induced genes [52]. In the current study, a total of 1122 miRNA target genes were identified in the sorghum genome based on the 69 previously known and 123 novel and unique candidate sequences of sorghum miRNAs that meet our filtering criteria (Table S8). To investigate the correlation of miRNA and target gene expression profiles, the gene expression profile of target genes was analyzed for differential expression as described above. We identified 149 target genes corresponding to 35 differentially expressed miRNAs (Table S9), which were potential *C. sublineolum* regulated target genes. The 62 potential miRNA target genes were differentially expressed in response to *C. sublineolum* (Fig. 5a and Table S10). Further observation indicated that a total of 23 miRNAs were identified as candidate miRNAs interacting with differentially expressed target genes (Fig. 5b and Table S10). A gene ontology (GO) functional classification analysis to understand the functions of the identified targeted genes resulted in a total of 37 GO terms including 12 cellular components, 15 biological process, and 10 molecular functions (Fig. 6). Of 12 cellular components, two most highly presented were GO terms describing parts of cells and structures associated with cells. Among the GO terms identified in biological process, two most frequent categories were metabolic process and cellular process. Catalytic activity and binding were identified as the most abundant molecular function.

## Discussions

This study was initiated to elucidate the molecular and genetic bases of sorghum resistance to the fungal pathogen *C. sublineolum* and to delineate the cellular and biological processes underlying fungal resistance. The fungus causes one of the most widespread diseases of sorghum. *C. sublineolum* is a typical hemibiotroph with an initial biotrophic stage that transitions into a necrotrophic phase. The later phase of the diseases involves extensive growth of secondary hyphae and enhanced fungal colonization of the host in susceptible genotypes. The current study on expression profiling was designed to cover the early biotrophic phase before the fungus extensively colonizes the plant, to avoid the secondary effects of pathogen colonization and extensive cellular damage that may result in activation of stress responsive genes. Our analyses of global mRNA and miRNA data shed light on pathways activated in response to anthracnose, identified potential genetic components, and cellular and metabolic processes that are important for plant responses to the pathogen. The discovery of genes and potential regulators such as miRNAs activated early after inoculation and their molecular and genetic characterization would provide the foundation for future molecular studies and, ultimately, for crop improvement. In particular, observations from this study can serve as the bases for additional genetic studies to find genes and/or loci with significant contribution to fungal resistance.

Two contrasting sorghum genotypes, highly resistant and susceptible genotypes with distinct responses were used in the global expression profiling experiment described here. TAM428 is highly susceptible to many races of *C. sublineolum* but appears to harbor low level of basal resistance and SC283 shows broad-spectrum resistance [41]. Based on mRNA profiles, we observe a significant reprogramming of gene expression early after fungal inoculation in both resistant and susceptible genotypes although the nature and functional groups vary. Interestingly, significant reprogramming of transcripts continues in the susceptible TAM428 even at 48 hpi while in the resistant genotype, a rapid and significant drop was observed in the number of genes that are up or down regulated. Rapid and extensive changes in gene expression were observed in the resistant line at 24 hpi but with significant decline at 48 hpi. This is consistent with continued fungal growth, and disease symptom observed in the susceptible but not in the resistant cultivar. A rapid increase in expression of genes encoding MAPKs, pentatricopeptide repeat proteins (PPR), photosynthesis and tetrapyrole biosynthesis genes were observed in the resistant which were repressed in the susceptible line.

Broadly, sorghum genes differentially expressed in response to *C. sublineolum* encode proteins in diverse biological functions including photosynthesis, carbohydrate metabolism, binding of chitin, secondary metabolism, N-metabolism, redox, biosynthesis of hormones and secondary metabolites, transport, and components of immune signaling. Many more genes in different functional groups were upregulated than down regulated in SC283. Interestingly, in the resistant line, there was a greater proportion of DEGs that encode proteins involved tetrapyrrole biosynthesis, photosynthesis, cell wall, and defense signaling. These processes are likely to impact resistance and severity of disease symptoms. By contrast, in TAM428, the proportion of downregulated DEGs are within the functional categories of N-metabolism, secondary metabolism, trapyrroles biosynthesis, hormone biosynthesis, TCA transformation and cell wall. The first three of these were clearly upregulated in SC283 suggesting that they may contribute to resistance. Tetrapyrroles contribute to detoxification of reactive oxygen species, the assimilation of nitrate and sulfate, respiration, photosynthesis, and programed cell death (PCD) [53]. Among these, PCD and accumulation of ROS are classical immune responses that affect plant resistance to pathogens. Interestingly, DEGs that correlated with resistance encode a range of proteins including pathogen recognition receptors, downstream components such as MAPKs, transcription factors, and genes involved in the synthesis of antimicrobial metabolites. The protein domain enrichment analyses uncovered chitin binding (IPR001002), and Lectin (IPR019825) [54] domain containing proteins were overrepresented in SC283. The chitin binding domain may occur in one or more copies and is thought to be involved in recognition or binding of chitin subunits, and degradation products of fungal cell walls that normally occurs during fungal infection of plants**.** Lectins are also carbohydrate-binding proteins which bind either glucose/mannose or galactose [55]. These domains are likely important in recognition of pathogen derived signals such as that of fungal chitin which is important for quantitative resistance triggered by chitin perception. Differences in pathogen recognition events early during infection may make significant differences in resistance as observed in SC283 which exhibits remarkable level of resistance to *C. sublineolum*.

Caution should also be exercised in the interpretation of the data and explaining the phenotypes of the two lines to specific pathogen induced genes. The two lines while showing very contrasting disease resistances also vary in many other traits. It is, however, clear that in the susceptible line, due to the elevated fungal growth many genes were induced especially at 48 hpi, some of which may mark a cellular perturbation rather than a specific immune response function. Additional genetic studies will be required to determine the important genetic players, among the DEGs, that directly contribute to resistance.

Mechanisms of sorghum resistance to *C. sublineolum* have been poorly studied especially at the molecular and biochemical levels. The immune response pathways that regulate resistance to this major pathogen is unclear. Despite the widespread and damaging nature of diseases caused by species in the genus *Colletotrichum* the host response pathways are understudied. Although both quantitative and race specific resistances have been described, neither the specific components of the pathogen nor the specific genes in the host have been determined. Some of the DEGs identified may contribute to resistance through modulation of HR, accumulation of reactive oxygen species, enhancement of cell wall, and biosynthesis of antimicrobial compounds. The increased expression of genes encoding some of the classical PR proteins such as chitinases, in the susceptible genotype do not appear to explain the disease responses differences in the two lines. It is still possible that upregulation of sorghum chitinases may contribute to quantitative resistance in the susceptible background but are likely not sufficient to confer full resistance consistent with the disease responses and phenotypes of TAM428.

Further, components of plant immune response pathways are unclear in crop plants despite extensive studies in limited model systems. As such these genome wide mRNA and miRNA profiling data provide genes regulated during pathogen infection that can be pursued through genetic studies or are used as markers for tracking activation of immune responses. Interestingly, activation of some of these immune response genes such as PR proteins mark responses to infection rather than direct contribution to resistance. MAPK proteins have been implicated in defense and growth regulation in Arabidopsis and other plant systems with very limited studies in crop plants. Regardless of the particular pathway, plant immunity to fungal pathogens converge into common down-stream immune response such as oxidative burst, accumulation of phytoalexins and PR proteins [16]. Multiple studies have documented the biosynthesis of secondary metabolites such as the sorghum phytoalexin 3-Dexoxyantocyanidin in resistance to a range of fungal pathogens including *C. sublineolum* and molding fungi [24, 56].

In addition to various gene families and functional groups described in the preceding sections, a large number of genes encoding Pentatricopeptide-repeat proteins (PPR) were identified to be regulated during fungal infection. PPRs are a family of proteins commonly found in the plant kingdom that are distinguished by the presence of tandem degenerate PPR motifs (35-amino acid sequence motif) and by the relative lack of introns in PPR encoding genes [57]. Despite the large size of the protein family, genetic data suggest that there is little or no functional redundancy between PPR proteins in Arabidopsis. Genetic studies implicate some PPR genes to fungal resistance in Arabidopsis [58].

This report also studied fungal induced microRNA and their potential regulatory role in sorghum which has not been done previously. Non-coding RNAs are components of gene regulatory networks in eukaryotic genomes. Among these, ∼20–30 nt small RNAs molecules, have been implicated as key regulators of expression and function [59]. miRNAs play an important role in plant development and disease resistance processes through post-transcriptional regulation by targeting mRNAs for cleavage or repressing translation [60, 61]. Translocation of small RNAs between interacting partners regulate host gene expression and suppress plant immunity. In one example, the fungal pathogen *B. cinerea* suppresses host immunity through translocation of microRNAs [33]. Conversely, host induced gene silencing have been considered to interfere with pathogen/parasite virulence mechanisms and is proposed for application to engineer resistance to fungal pathogens, nematodes and insect pests [62]. Some studies report miRNAs involved in abiotic stress responses of sorghum [40, 63, 64] but there were no studies in host responses to pathogen infection in sorghum. In the recent release of miRBase (version 21), a total of 241 miRNAs have been identified based on the sorghum reference genome (BTX623, Sorbi1) [65]. However, the recent sorghum genome sequences (BTX623, Sbicolor_313_v3.1) [66] provides an opportunity for accurate identification of novel miRNAs across different sorghum cultivars. The number of novel miRNAs discovered is relatively low, similar to previous reports including a study on miRNA sequencing of tomato in response to drought and chilling stress [67, 68], and high-throughput sequencing of Arabidopsis miRNAs [69].

Our study identified miRNA and their corresponding target gene that are regulated by *C. sublineolum.* The identified target genes were diverse and include genes that encode for major immune response regulators such as pattern recognition receptors and nucleotide binding/leucine-rich repeat (NBs-LRR) resistance proteins. Previously, miRNAs and secondary siRNAs have been implicated in NBs-LRR gene regulation and pathogen resistance in the *Solanaceae* [70]. Interestingly, small RNAs also regulate immunity during plant growth to prevent autoimmune regulation of NBs-LRR genes [71]. Similarly, in Arabidopsis miRNA have been implicated in regulating PAMP triggered immunity (PTI). The miRNA effector protein, Argonaute1, is implicated in PTI responses including PAMP-induced callose deposition, gene expression, and seedling growth inhibition [72]. In the same study, a number of differentially expressed miRNAs that are differentially regulated by the bacterial PAMP flg22 were identified. Functional studies demonstrated that *miR160a* positively regulate PAMP-induced immune responses, whereas *miR398b* and *miR773* suppress PAMP-induced callose deposition and disease resistance [72]. These two reports demonstrate the role of miRNAs in regulating major immune response pathways. Similar knowledge on the role of miRNAs in regulating immune response gene expression in sorghum and other related crop plants have been limited.

## Conclusions

Transcriptional activation of genes encoding a battery of molecules is a critical component of plant immunity. Timely activation of immune responses makes the difference between resistance and susceptibility. This study shed light on global gene expression patterns cellular processes, metabolic pathways, and immune response genes that correlate with resistance. Expression of genes in plant photosynthetic pathways and tetrapyrrole synthesis of secondary metabolites were associated with host resistance responses to this fungus. Genes encoding protein families such as PPRs, LRR domain proteins, chitin and lectin binding correlated with resistance. Similarly, a rapid increase in expression of MAPKs, photosynthesis and tetrapyrole biosynthesis genes in the resistant genotype was observed. Finally, this study catalogued fungal induced miRNAs with a likely regulatory role in sorghum resistance, and their corresponding targets. These results will guide future genetic studies to validate the most important determinants of fungal resistance and also pave the way for molecular and genetic studies to dissect mechanisms of resistance to *C. sublineolum*.

## Methods

### Sorghum lines

The sorghum genotypes SC283 and TAM428 were previously described to show enhanced resistance and susceptibly to the *C. sublineolum*, respectively [10, 73]. SC283 shows broad-spectrum resistance to about 10 strains of Cs tested while TAM428 was susceptible to the same strains, and detailed molecular and phenotype characterization of the two genotypes is described [74].

### Fungal media preparation and inoculation

For disease assays, fungal spores of *C. sublineolum* strain *Csgl2* originally collected in Indiana, USA, was cultured on potato dextrose agar (PDA). The culture medium was autoclaved for 15 min at 121 °C prior to initiating fungal culture. The culture was maintained under continuous fluorescence light. Conidia were harvested from 15-day old cultures and used to inoculate 3 weeks-old plants of SC283 and TAM428. Plants were mock sprayed or inoculated with a conidial suspension at a concentration of 1 × 10^6^ spores/mL. Inoculated plants were kept under 70% relative humidity for about 48 h and transferred to the greenhouse with an overhead misting for further observation of disease development.

### RNA isolation and RNA-seq library preparation

Leaf tissue samples (100 mg) for RNA isolation were collected from each genotype at 0, 24 and 48 h post inoculation (hpi). Total RNA was isolated as described in Spectrum™ Plant Total RNA kit protocol with on-column DNase digestion (Sigma-Aldrich, USA). RNA samples were purified using RNA clean and concentration TM-25 (ZYMO RESEARCH) and the quality was determined both by NanoDrop and Agilent 2100 Bioanalyzer. A total RNA (~ 3 μg) for each sample was used to prepare mRNA-seq library according to TrueSeq RNA Sample Prep Kit protocol (Illumina). Library quality control and quantification were performed with an Experion DNA 1 K Chip (Bio-Rad) and a Qubit fluorometer (Invitrogen), respectively. For each library, more than 100 million, 100-bp paired-end sequences were generated (Table 1) using an Illumina HiSeq 2500.

### Sequence quality control

Adapters and low-quality reads containing uncalled bases were removed. The quality of RNA-seq data was assessed by FastQC [75] and the low-quality reads were removed by Fastx-toolkit [76]. The TopHat2 software [77] was used following quality control of the sequence to align the pair end RNA-seq reads against sorghum (BTx623 genotype) reference genome version 3.1 [66] accessed on Phytozome version 10 [78]. TopHat2 alignment parameters were set to allow a maximum of two mismatches to exclude read mapping to more than one position on the reference. Moreover, only reads for which both pairs successfully aligned were considered. The HTSeq python tool [79] were used to process the read counts. For sRNA library construction, 1 μg of total RNA were used for small RNA library with 50 nt selection size using polyacrylamide gel electrophoresis (PAGE). The library was sequenced using Illumina Genome Hiseq 2000.

### Hierarchical clustering and dispersion analysis

To assess variability among samples, we performed hierarchical clustering and dispersion analysis based on biological coefficient of variation (BCV). Hierarchical clustering was performed based on Euclidean distances. Dispersion analysis was conducted using top 2000 values using an EdgeR package [49] in R software [50].

#### Differential gene expression analysis

Differential expression analyses were performed with EdgeR package [49] in R software [50] using empirical Bayesian methods. To filter out weakly expressed genes, only those genes with a minimum expression level of 1 RPKM (reads per kilobase per million) in three replicates were included in the analysis. Genes with a log fold change (LogFC) above one (two-fold change), false discovery rate (FDR) and *P*-values below 0.05 were considered differentially expressed between conditions.

### Functional classification of differentially expressed genes

To evaluate potential function of the large number of genes with differential expression in response to *C. sublineolum*, all DEGs, both from SC283 and TAM428, were combined and classified using InterProScan [80] and MapMan software [81]. For MapMan analysis, all gene identification label was converted into Sbicolor_79 label based on Sbicolor 3.1 annotation files (PhytozomeV10: Sbicolor_313_v3.1. synonym). Then, we used agriGO and ReviGO [82, 83] to identify putative biological functions and biochemical pathways for differentially expressed genes (DEGs) and find statistically overrepresented gene ontology (GO) terms.

### Categorization of sorghum protein functional domains

The sorghum protein domains and protein family analyses were conducted mainly on the basis of Pfam domains [84] and PANTHER families [85] of proteins with sorghum genome sequence annotation file (PhytozomeV10: Sbicolor_313_v3.1). To categorize gene expression patterns for protein function domains, genes with a LogFC above 5 (32 folds change) and false discovery rate (FDR) and *P*-values < 0.05 were selected.

### Validation of gene expression by qRT-PCR

Quantitative RT-PCR (qRT-PCR) was used to validate the RNA-seq data. A total of 12 genes were selected for analyses of gene expression. The reverse transcription and RT-PCR were carried out using qRT-PCR SYBR® Kits. RNA was isolated using the Spectrum™ Plant Total RNA Kit with on-column DNaseI digestion (Sigma-Aldrich, St. Louis, USA) for removal of residual genomic DNA. Reverse transcription was conducted in a 25 μl mixture containing 5 μl reaction buffer (5x), 1 μl RT enzyme, 2 μl of dT, 1 μl dNTPs and 16 μl RNA sample. The mixture was incubated at 42 °C for 1 h, followed by inactivation of the reverse transcriptase at 90 °C for 10 min. The process resulted in 25 μl of cDNA and 150 μl DEPC water was added making 175 μl total volume cDNA for each sample. The diluted cDNA was used as templates for expression profiling of selected genes. Real-time PCR was performed on CFX 96 Real-Time System (Bio-Rad Laboratories, USA), in a 15 μl of mixture containing 7.5 μl SYBR Green master mix (2x), 3.5 μl ddH_2_O, 1.0 μl of forward and reverse primer (10 μM) and 2 μl cDNA template. Similar reaction mix was used for the candidate genes. The PCR program used was 95 °C for 3 min, 40 cycles of 95 °C for 10 s, 55 °C for 30 s, and 72 °C for 30 s, followed by melt curve 65.0 to 95.0 °C, increment 0.5 °C, 5 s for plate read. For data analysis, the mean Ct value of the target gene was normalized to the average Ct value of sorghum actin gene using the ΔCt method implemented in the CFX manager software (Bio-Rad Laboratories, USA).

### miRNA prediction

We applied an informatics pipeline for filtering plant miRNAs from the complete set of small RNAs. A total of 228,228,937 distinct small RNAs reads were analyzed using the pipeline from 12 mRNA libraries prepared both samples that were mock or 48 hpi after *C. sublineolum* inoculation. All small RNAs reads were filtered following the steps described (Figure S3). First, adaptors and low-quality reads were removed using FASTX-Toolkit [86]. Second, structural RNAs like tRNAs and rRNAs were removed. Third, selection of RNA read size between 18 and 28 nucleotides (nts) were conducted. Fourth, low abundance small RNAs were removed by retaining those > 10 transcripts per million, in at least one of the twelve libraries. Furthermore, *C. sublineolum* genome reads, and highly repetitive small RNAs (those with hits to genome > 20) were removed, and 121,338 small RNAs were retained for miRDeep-P [87] miRNA prediction. To keep consistency in miRNAs identification, all small RNA libraries were separately processed based on the above pipeline. Newly identified miRNAs were considered as candidate only when detected consistently across the three replicates of the libraries with similar treatment in SC283 or TAM428. In order to further verify our predicted miRNAs, highly similar homologs in miRBase V21 were identified using Sgemel [88]. The miRNAs that passed the processing filter were considered new.

### miRNA expression profiling and target prediction

The identified miRNAs were analyzed for differential expression with EdgeR package [49] in R software [50] using empirical Bayesian methods. miRNA targets were predicted using CleaveLand with penalty score ≤ 5 [89]. A gene ontology (GO) functional classification analysis was employed to further predict the functions of the identified targeted genes using AgriGO [82].

## Supplementary information

Additional file 1: **Table S1.** Receptor like Kinase (RLK) family genes differentially expressed in TAM428 and SC283 genotypes of sorghum. (XLSX 10.9 kb)

Additional file 2: **Table S2.** Genes encoding NBS-LRR proteins differentially expressed in susceptible and resistant genotypes of sorghum. (XLSX 16.1 kb)

Additional file 3: **Table S3.** Differentially expressed genes encoding chitinases in susceptible and resistant genotypes of sorghum. (XLSX 11.0 kb)

Additional file 4: **Table S4.** WRKY genes differentially expressed in susceptible and resistant genotypes of sorghum. (XLSX 12.8 kb)

Additional file 5: **Table S5.** MAPK genes differentially expressed in susceptible and resistant genotypes of sorghum. (XLSX 12.6 kb)

Additional file 6: **Table S6.** Pentatricopeptide (PPR) genes differentially expressed in susceptible and resistant genotypes of sorghum. (XLSX 17.8 kb)

Additional file 7: **Table S7.** Novel mature miRNA loci and precursor sequences in sorghum genome. (XLSX 33.4 kb)

Additional file 8: **Table S8.** List of all miRNA target genes identified in the sorghum genome. (XLSX 129 kb)

Additional file 9: **Table S9.**Target genes identified, their classification and identification of the function of gene products with different databases. (XLSX 27.5 kb)

Additional file 10: **Table S10.** List of differentially expressed miRNA target genes and their corresponding miRNAs in sorghum. (XLSX 15.2 kb)

Additional file 11: **Figure S1.** Validation of RNA-seq data through qRT-PCR analyses of selected differentially expressed genes in response to *C. sublineolum*. Gene expression data are normalized by the comparative cycle threshold method with the sorghum *Actin2* as the internal control. Data represent means ± SD (*n* = 3). The graph shows both gene expression based on RNA-seq data and qRT-PCR. (PPTX 798 kb)

Additional file 12: **Figure S2**. Metabolic changes after fungal infection in (A) SC283 and (B) TAM428 visualized by Mapman. Mapman functional categories were generated for genes with altered expression before and after inoculation. Genes significantly up- and down-regulated in infected leaves relative to mock-inoculated leaves are presented in red and blue, respectively. Scale bars display Log2-fold changes. Only significant changes are displayed for CHO, carbohydrates; OPP, oxidative pentose phosphate pathway; TCA, tricarboxylic acid cycle. (PPTX 433 kb)

Additional file 13: **Figure S3.** Small RNA analysis pipeline and prediction results for candidate miRNAs in sorghum. Known miRNAs were identified using mirDeep and novel miRNA were predicted using mirDeep-P based on four automated filtering steps and a final comparison to annotated miRNAs in miBase. The filters are described in the main text and summarized in the flowchart on the left. On the right are the results of the application of the pipeline in sorghum. Numbers in red indicate those predicted novel microRNAs by mirDeep-P. (PPTX 34.1 kb)

## Data Availability

The RNA-seq and microRNA-seq data have been submitted to NCBI https://www.ncbi.nlm.nih.gov/bioproject/PRJNA667277**,** under the accession number PRJNA667277.

## Abbreviations

qRT-PCR: Quantitative real-time PCR
nt: Nucleotide
GO: Gene ontology
miRNAs: microRNAs
DEGs: Differentially expressed genes
FDR: False discovery rate
LogFC: Log fold change
