# Supplementary figures and images for: Global mRNA and microRNA expression dynamics in response to anthracnose infection in sorghum
Source: BMC Genomics. 2020 Nov 3;21:760. doi: 10.1186/s12864-020-07138-0 (PMC7641857; doi:10.1186/s12864-020-07138-0)

## Slide 1
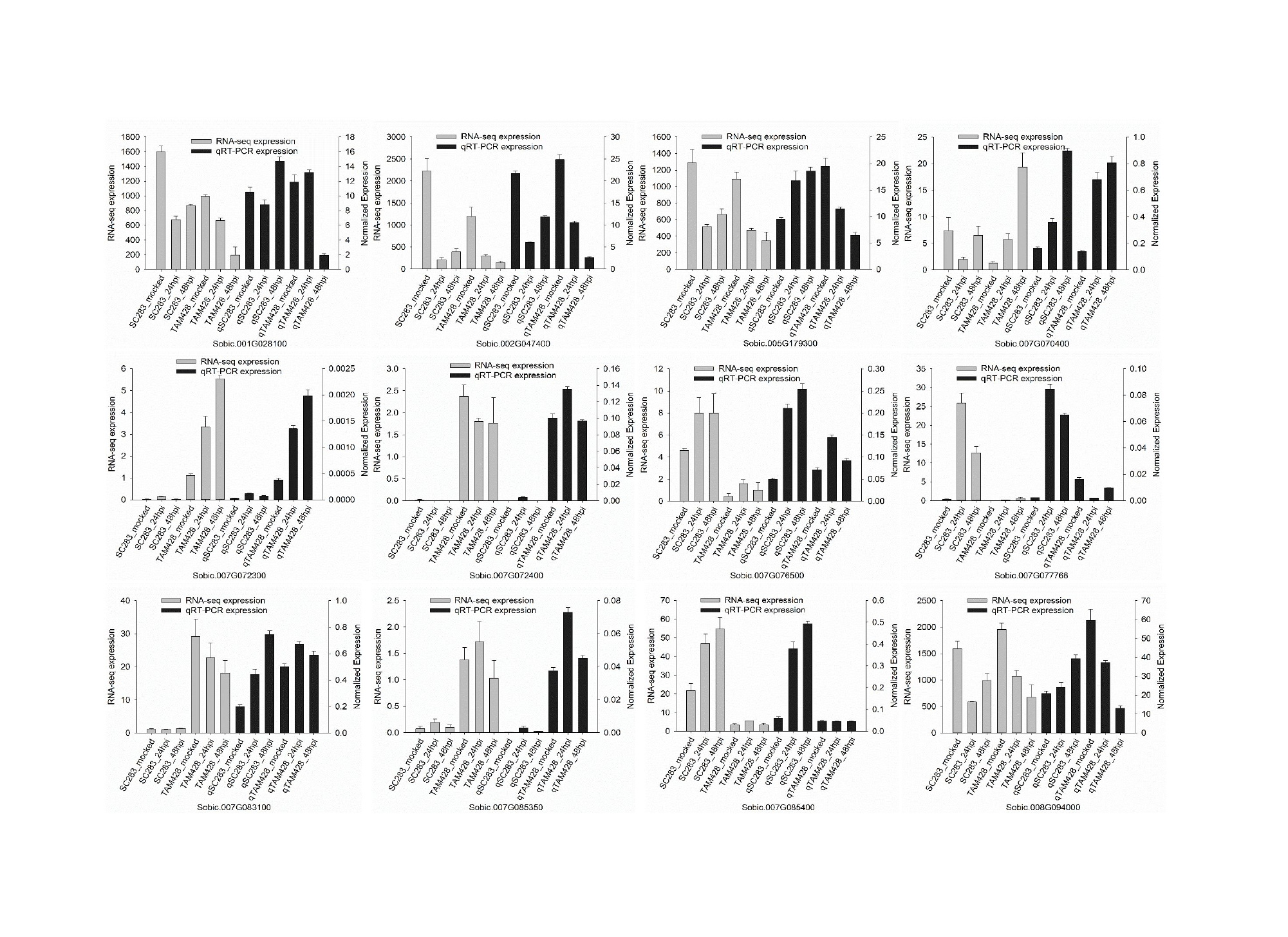

Supplement: Supplementary file 11 — Figure S1. Validation of RNA-seq data through qRT-PCR analyses of selected differentially expressed genes in response to C. sublineolum. Gene expression data are normalized by the comparative cycle threshold method with the sorghum Actin2 as the internal control. Data represent means ± SD (n = 3). The graph shows both gene expression based on RNA-seq data and qRT-PCR. (PPTX 798 kb) [file 12864_2020_7138_MOESM11_ESM.pptx]

## Slide 1
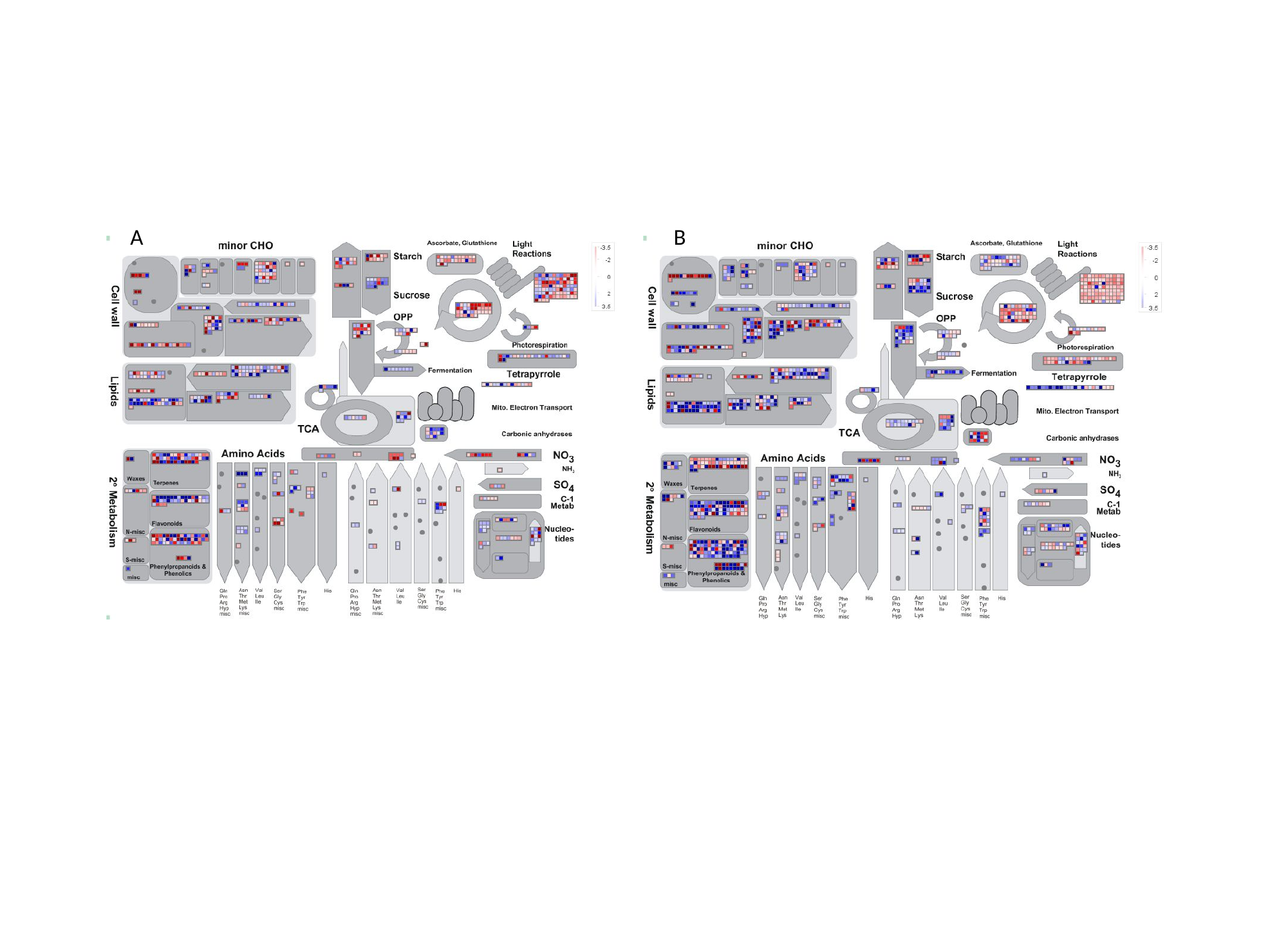

A
B

Supplement: Supplementary file 12 — Figure S2. Metabolic changes after fungal infection in (A) SC283 and (B) TAM428 visualized by Mapman. Mapman functional categories were generated for genes with altered expression before and after inoculation. Genes significantly up- and down-regulated in infected leaves relative to mock-inoculated leaves are presented in red and blue, respectively. Scale bars display Log2-fold changes. Only significant changes are displayed for CHO, carbohydrates; OPP, oxidative pentose phosphate pathway; TCA, tricarboxylic acid cycle. (PPTX 433 kb) [file 12864_2020_7138_MOESM12_ESM.pptx]
