# Supplementary material for: Global mRNA and microRNA expression dynamics in response to anthracnose infection in sorghum
Source: BMC Genomics. 2020 Nov 3;21:760. doi: 10.1186/s12864-020-07138-0 (PMC7641857; doi:10.1186/s12864-020-07138-0)
Supplement: Supplementary file 13 — Figure S3. Small RNA analysis pipeline and prediction results for candidate miRNAs in sorghum. Known miRNAs were identified using mirDeep and novel miRNA were predicted using mirDeep-P based on four automated filtering steps and a final comparison to annotated miRNAs in miBase. The filters are described in the main text and summarized in the flowchart on the left. On the right are the results of the application of the pipeline in sorghum. Numbers in red indicate those predicted novel microRNAs by mirDeep-P. (PPTX 34.1 kb) [file 12864_2020_7138_MOESM13_ESM.pptx]

## Slide 1
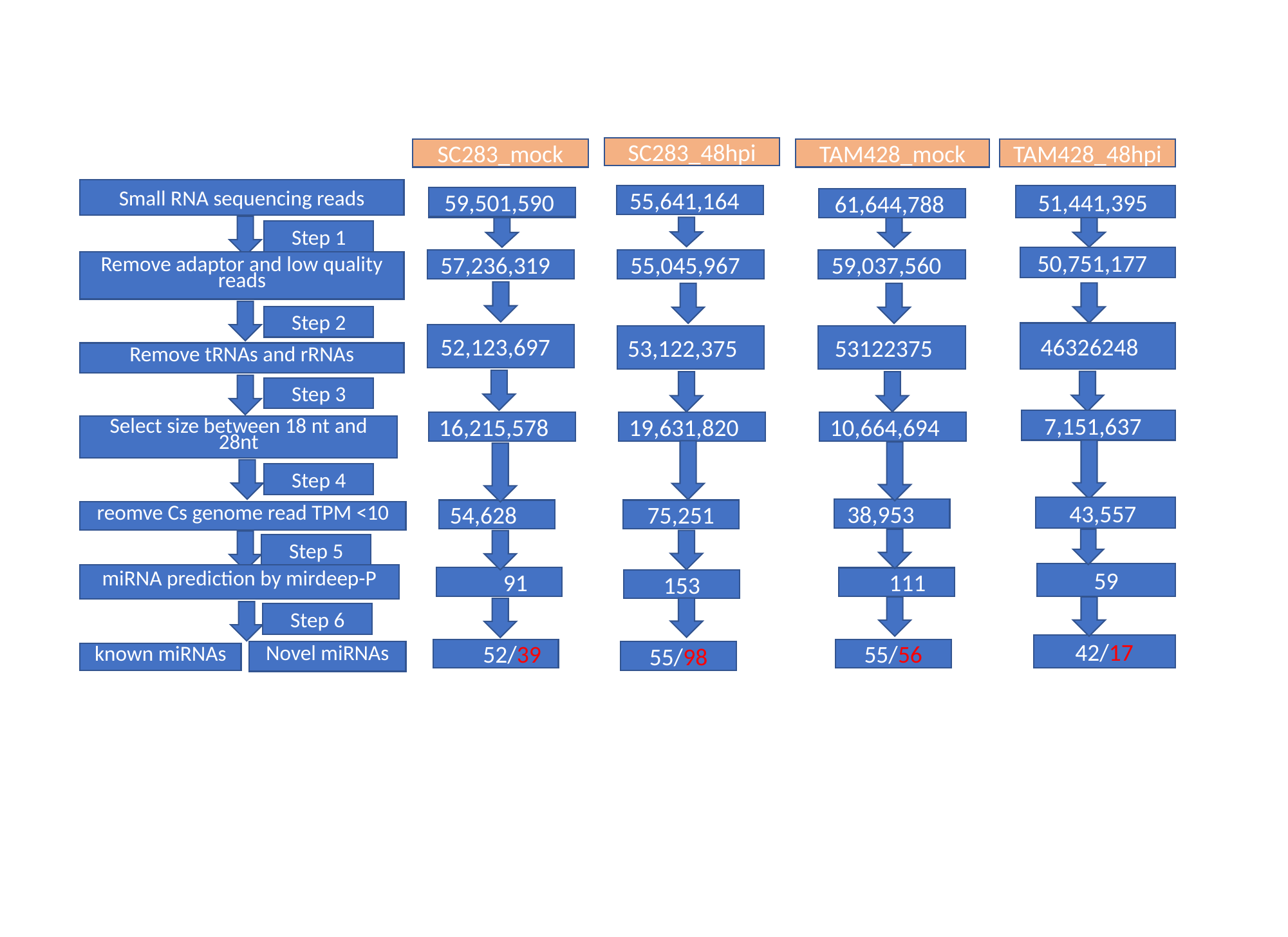

SC283_48hpi
TAM428_48hpi
TAM428_mock
SC283_mock
55,641,164
51,441,395
59,501,590
59,501,590
61,644,788
50,751,177
55,045,967
59,037,560
57,236,319
46326248
52,123,697
53122375
53,122,375
7,151,637
19,631,820
10,664,694
16,215,578
43,557
38,953
54,628
75,251
59
 91
 111
153
Small RNA sequencing reads
Step 1
Remove adaptor and low quality reads
Step 2
Remove tRNAs and rRNAs
Step 3
Step 4
reomve Cs genome read TPM <10
Select size between 18 nt and 28nt
Step 5
Novel miRNAs
known miRNAs
miRNA prediction by mirdeep-P
Step 6
42/17
 52/39
55/56
55/98
